# Predominance of III/ST19 and Ib/ST10 Lineages With High Multidrug Resistance in Fluoroquinolone-Resistant Group B *Streptococci* Isolates in Which a New Integrative and Conjugative Element Was Identified

**DOI:** 10.3389/fmicb.2020.609526

**Published:** 2021-01-25

**Authors:** Kankan Gao, Chunyan Gao, Lianfen Huang, Xiaoshan Guan, Wenjing Ji, Chien-Yi Chang, David J. McIver, Qiulian Deng, Huamin Zhong, Yongqiang Xie, Lei Deng, Fei Gao, Lanlan Zeng, Haiying Liu

**Affiliations:** ^1^Clinical Laboratory, Guangzhou Women and Children's Medical Center, Guangzhou Medical University, Guangzhou, China; ^2^Clinical Laboratory, Tangshan Municipal Women and Children's Hospital, Tangshan, China; ^3^Department of Pharmacy Administration and Clinical Pharmacy, School of Pharmacy, Center for Drug Safety and Policy Research, Xi'an Jiaotong University, Xi'an, China; ^4^School of Dental Sciences, Newcastle University, Framlington Place, Newcastle upon Tyne, United Kingdom; ^5^Global Health Group, Institute for Global Health Sciences, University of California, San Francisco, San Francisco, CA, United States

**Keywords:** fluoroquinolone, group B *Streptococcus*, multi-drug resistance, antimicrobial resistance, neonatal invasive infection, maternal colonization, integrative conjugative element

## Abstract

Fluoroquinolone (FQ)-resistant Group B *Streptococcus* (GBS) has been reported with considerable cross-resistance, worsening the crisis of multidrug-resistant (MDR) GBS in clinical settings. However, national epidemiological data on FQ-resistant GBS in mainland China have not been well-characterized. This study aimed to determine the prevalence of FQ resistance among GBS from neonatal invasive infections and maternal colonization in northern and southern China, to investigate the serotyping, multilocus sequence typing, and antibiotic cross-resistance, and to characterize the mutations in *gyrA* and *parC* genes in quinolone resistance-determining region (QRDR). In order to provide a comprehensive view of the location and structure of resistance genes, whole-genome sequencing on III/ST19 MDR isolates were performed. Among 426 GBS, 138 (32.4%) were FQ resistant, with higher prevalence in northern China than in southern China in both neonates (57.8%, 37/64 vs. 21.7%, 39/180) and pregnant women (50.9%, 29/57 vs. 26.4%, 33/125). Serotypes were distributed as III (48.5%), Ib (39.9%), V (6.5%), and Ia (5.1%). Sequence types were mainly ST19 (53.6%) and ST10 (39.1%), followed by ST12 (1.4%), ST17 (1.4%), ST23 (1.4%), and 0.7% each of ST27, ST188, ST197, and ST597. ST19 isolates were more prevalent in southern China than in northern China in both neonates (64.1%, 25/39 vs. 27.0%, 10/37) and pregnant women (81.8%, 27/33 vs. 41.4%, 12/29), whereas ST10 isolates were more common in northern China than in southern China in both neonates (64.9%, 24/37 vs. 20.5%, 8/39) and pregnant women (58.6%, 17/29 vs. 15.2%, 5/33). Serotype III isolates were mainly ST19 (89.6%, 60/67), while Ib isolates were largely ST10 (94.5%, 52/55). Sequencing data revealed several mutations in QRDR, including Ser81Leu in *gyrA* (99.2%, 130/131), Ser79Phe or Tyr in *parC* (76.2%, 48/63), and a previously unreported Ile218Thr and Ile219Phe double mutation pattern (49.2%, 31/63) in *parC*. ST10 isolates were associated with Ser79Phe (84%, 21/25), while ST19 isolates were limited to Ser79Tyr (95.7%, 22/23). A new integrative and conjugative element (ICE) harboring *tetM* and *gyrA* genes was identified in a III/ST19 isolate. This study investigates the molecular characteristics of FQ-resistant GBS in northern and southern China, emphasizing the need for continuous surveillance geographically and further research to characterize the mechanisms of ICE transfer.

## Introduction

Group B *Streptococcus* (GBS), also known as *Streptococcus agalactiae*, commonly colonizes the human gastrointestinal and genitourinary tracts (CDC, 2010) and has been recognized as the leading contributor to adverse maternal and neonatal outcomes (Seale et al., 2017; Zhang et al., 2019). Intrapartum use of prophylactic antibiotics in pregnant women with GBS colonization has been shown to be very successful in reducing the incidence of early-onset GBS disease (CDC, 2010), but this strategy has not been fully adopted in mainland China. While GBS remains largely susceptible to penicillin, penicillin susceptibility reduced GBS isolates have been recently described (Kimura et al., 2008; Longtin et al., 2011; Moroi et al., 2019). Considering that the prevalence of penicillin allergy is as high as 11.5% (Albin and Agarwal, 2014), the alternative clindamycin has a resistance rate of up to 33% (Teatero et al., 2017), and the other alternative erythromycin is no longer recommended (Puopolo et al., 2019; Opinion, 2020). Fluoroquinolone (FQ) has emerged as an important alternative for GBS treatment, but worryingly, GBS resistance to FQ has already been reported (Wu et al., 2008; Piccinelli et al., 2015; Hays et al., 2016). In mainland China, most FQ-resistant GBS strains have been isolated from urine and wounds (Wang et al., 2013). Little is known regarding the molecular epidemiology of FQ-resistant GBS isolated from neonatal invasive infections and maternal colonization nationwide. Moreover, FQ-resistant GBS isolates displayed widespread antibiotic cross-resistance (Hays et al., 2016), worsening the crisis of multidrug-resistant (MDR) GBS in clinical settings. The horizontal transfer of mobile genetic elements (MGEs) via conjugation has been suggested as an important determinant of the dissemination of antibiotic resistance genes (Von Wintersdorff et al., 2016).

Therefore, in this study, we investigated the molecular subtyping, antibiotic resistance characteristics of FQ-resistant GBS, and mutations of the *gyrA* and *parC* genes in quinolone resistance-determining region (QRDR). Whole genome sequencing analysis on MDR isolates were performed for identifying the potential MGEs in these FQ-resistant GBS isolates.

## Materials and Methods

### Clinical Isolates

Neonatal invasive GBS isolates were obtained from a nationwide multicenter study involving 18 urban tertiary hospitals across 16 provinces in China, from January 2015 to December 2017. Neonatal invasive cases were defined as illnesses among infants <3 months of age with clinical symptoms, including but not limited to fever, breathing problems, fussiness, cyanosis, seizures, limpness, or stiffness. Specimens were obtained from blood and/or cerebrospinal fluid, which were GBS positive by culture. Early-onset diseases were defined as cases occurring within 0–6 days of birth, and late-onset diseases were defined as cases happening within 7–89 days of birth.

The eligibility criteria for maternal participation in this study included all pregnant women aged >18 years between 35 and 37 weeks gestational age at the Tangshan Municipal Women and Children's Hospital, Tangshan (northern China), and the Guangzhou Women and Children's Medical Center, Guangzhou (southern China). Specimens were collected from lower vaginal and rectal swabs.

All clinical data were acquired from electronic databases of the hospital information management systems at each site. Records were considered as the same episode if specimens were taken within 7 days and showed resistance to the same antibiotics and were thus merged accordingly to form a single record. Sampling, laboratory procedures, and identification of GBS were performed as previously described (Ji et al., 2019; Li et al., 2019).

### Antimicrobial Susceptibility Testing

Antibiotic resistance profiles were determined by minimum inhibitory concentrations (MICs) using VITEK 2 COMPACT microbiology system (BioMérieux, France). Breakpoint interpretation was done according to the criteria of the Clinical and Laboratory Standards Institute (CLSI), 2020 Edition (CLSI, 2020). A double-disk agar diffusion test (D-test) was performed to detect the inducible macrolide–lincosamide–streptogramin B phenotype. *Streptococcus pneumoniae* ATCC 49619 and *Staphylococcus aureus* ATCC25923 were used as quality controls. Antimicrobial agents containing erythromycin, clindamycin, tetracycline, levofloxacin, penicillin, ampicillin, vancomycin, tigecycline, and linezolid were tested against all isolates. An FQ-resistant isolate was defined as a levofloxacin minimum inhibitory concentrations (MIC) >8 mg/L. An MDR isolate was defined as resistance to at least two of the antimicrobial agent classes above in addition to levofloxacin.

### Molecular Subtyping

Capsular serotyping, multilocus sequence typing (MLST), and clonal complex (CC) assigning were conducted using methods as previously described (Ji et al., 2019).

### Detection of Resistance Genes and QRDR Mutations

Resistance genes for clindamycin (*lnuB*), erythromycin (*ermB*), and tetracycline (*tetO* and *tetM*) were detected by PCR as previously described (Gao et al., 2018; Li et al., 2019). Identification of the *gyrA* and *parC* genes in QRDR mutations was performed according to Hays et al. (2016).

### PacBio Library Construction and Sequencing

GBS DNA was extracted from cultured isolates using the Qiagen Genomic tip 100/G kit (Qiagen, Germany) to facilitate long-fragment extraction. Quality and fragment-length distributions were assessed using the Qubit fluorometer (Thermo Fisher Scientific, USA) and TapeStation (Agilent, USA). Full-length complementary DNA (cDNA) was prepared using a SMARTer™ PCR cDNA Synthesis Kit (Takara Japan). Filtered full-length cDNA were subjected to reamplification, end repair, single-molecule real-time (SMRT) adapter ligation and exonuclease digestion. To obtain the longest possible SMRTbell libraries for sequencing, an additional size-selection step was performed using the PippinHT PFGE system (Sage Science, USA). The quantity and quality of the SMRTbell libraries were evaluated using the high-sensitivity double-stranded (dsDNA) kit and Qubit fluorometer and DNA 12000 kit on a 2100 Bioanalyzer (Agilent, USA). Subsequently, each library was sequenced using a single SMRT cell on the PacBio RSII sequencing system. The complete nucleotide sequence of the strain BJ01 was submitted to the National Center for Biotechnology Information (NCBI) GenBank and assigned accession number CP059383.

### Genome Alignments and Sequence Analysis

Integrative and conjugative element (ICE) sequence alignments and comparison were performed using Easyfig (Sullivan et al., 2011). ICE sequence similarities were searched on the ICEberg server (Bi et al., 2012). The origin site of DNA transfer (*oriT*) and type IV secretion system (T4SS) of the ICE were searched on the oriTfinder server and its database (Li et al., 2018). The distribution of the antibiotic resistance genes present in the comprehensive antibiotic resistance database (Liu and Pop, 2009) within the genomes of the strain BJ01 was investigated using custom scripts based on the BLAST and HMMer algorithms.

### Statistical Analysis

Chi-squared or Fisher's exact test (two-tailed) were used for comparison of categorical variables. Statistical analyses were performed using SPSS software 21.0 (IBM, USA). A *P* < 0.05 was considered statistically significant. Antibiotic susceptibility data were extracted using WHONET 5.6 software (WHO Collaborating Center for Surveillance of Antimicrobial Resistance, USA).

## Results

### Epidemiologic, Clinical, and Geographical Characteristics of FQ-Resistant GBS

In this study, 426 unduplicated GBS strains were obtained, including 244 neonatal invasive strains and 182 maternal colonization strains, of which 76 (31.1%) and 62 (34.1%) were resistant to levofloxacin, respectively, with the overall FQ resistance of 32.4% (*n* = 138). All 426 strains were sensitive to penicillin, ampicillin, vancomycin, tigecycline, and linezolid, and no penicillin susceptibility reduced GBS isolate was found. However, high prevalence of resistance to tetracycline (81.4%), erythromycin (74.4%), and clindamycin (68.5%) was observed.

The 244 GBS isolates from neonates (aged 4.0 days; interquartile range, 0, 22.0 days), including 106 early-onset diseases and 138 late-onset diseases, were geographically divided into northern and southern China. Among them, FQ resistance in northern China (57.8%) was significantly higher than that in southern China (21.7%, *P* < 0.001; Table 1). Of the 182 GBS isolates collected from maternal colonization (aged 30.6 ± 4.6 years), the FQ resistance in northern China (50.9%) was also significantly higher than that in southern China (26.4%, *P* = 0.001; Table 1).

**Table 1:** Epidemiological, clinical, and geographical characteristics of FQ-resistant GBS.

**Categories**	**All isolates (*n*)**	**FQ-resistant GBS (*n*, %)**	***P*-value**
Neonatal invasive infection^a^			
Sex [n (%)]			0.236
Male	102	36 (35.3)	
Female	142	40 (28.2)	
Diseases [n (%)]			0.051
Early-onset disease	106	40 (37.7)	
Late-onset disease	138	36 (26.1)	
Specimens [n (%)]			0.569
Blood only	135	40 (29.6)	
CSF	109	36 (33.0)	
Regions [n (%)]			<0.001
Northern China	64	37 (57.8)	
Southern China	180	39 (21.7)	
Maternal colonization^b^			
Regions [n (%)]			0.001
Northern China	57	29 (50.9)	
Southern China	125	33 (26.4)	
Total	426	138 (32.4)	-

*FQ, fluoroquinolone; GBS, Group B Streptococcus; CSF, cerebrospinal fluid*.

a *Northern China: Tsinghua University Hospital (Beijing), Tianjin Central Hospital of Gynecology Obstetrics (Tianjin), Tangshan Municipal Women's and Children's Hospital (Tangshan), Shengjing Hospital, China Medical University (Shenyang), General Hospital, Ningxia Medical University (Yinchuan), Maternal and Child Health Care Hospital of Uygur Autonomous Region (Urumqi), and the First Affiliated Hospital of Xi'an Jiaotong University (Xi'an); Southern China: Guangzhou Women and Children's Medical Center (Guangzhou), Guangdong Women and Children's Hospital Guangzhou), Nanjing Maternity and Child Health Care Hospital (Nanjing), Women's Hospital, Zhejiang University (Hangzhou), Children's Hospital of Fudan University (Shanghai), Changsha Hospital for Maternal and Child Health (Changsha), and Hubei Maternal and Child Health Hospital (Wuhan)*.

b *Northern China: Tangshan Municipal Women's and Children's Hospital (Tangshan); Southern China: Guangzhou Women and Children's Medical Center (Guangzhou)*.

### Serotypes, Sequence Types, and Clonal Complexes

Among the 138 FQ-resistant isolates, serotypes were distributed as III (48.5%), Ib (39.9%), V (6.5%), and Ia (5.1%). ST19 (53.6%) and ST10 (39.1%) were the dominant sequence types, followed by ST12, ST17, and ST23, each accounting for 1.4%, and ST27, ST188, ST197, and ST597, accounted for 0.7% each. ST19 isolates were more prevalent in southern China than those in northern China both in neonates (64.1%, 25/39 vs. 27.0%, 10/37; *P* = 0.001) and pregnant women (81.8%, 27/33 vs. 41.4%, 12/29; *P* = 0.001), whereas ST10 isolates were more common in northern China than those in southern China both in neonates (64.9%, 24/37 vs. 20.5%, 8/39; *P* < 0.001) and pregnant women (58.6%, 17/29 vs. 15.2%, 5/33; *P* < 0.001; Table 2). The majority of sequence types was categorized as CC19 (55.1%) and CC10 (41.3%).

**Table 2:** Serotypes, sequence types, and mutations in *gyrA* and *parC* genes of FQ-resistant GBS.

**Serotype/ST-CCs(n)**	**Sources (n)**		**Topoisomerase mutations**	
	**Neonatal invasive infection**	**Maternal colonization**	***gyrA***	***parC***
Ia(7)				
ST10-CC10(2)	N(1)	S(1)	Ser81Leu(1), Ser81Leu+Ala219Pro(1)	Ser79Phe+Ile218Thr+Ile219Phe(1), WT(1)
ST19-CC19(4)	–	N(1), S(3)	Ser81Leu(2), Ser81Leu+Ala219Pro(1), WT(1)	Ser79Tyr+Ile218Thr+Ile219Phe(1), Ser79Tyr(3), WT(2)
ST23-CC23(1)	S(1)	-	Ser81Leu(1)	WT(1)
Ib(55)				
ST10-CC10(52)	N(23), S(8)	N(17), S(4)	Ser81Leu(37), Ser81Leu+Ala219Pro(15)	Ser79Phe(16), Ile218Thr+Ile219Phe(5), Asn72Asp+Ser79Phe(1), Ser79Phe+Ile218Thr+Ile219Phe(3), WT(27),
ST12-CC10(1)	S(1)	-	WT(1)	WT(1)
ST19-CC19(1)	S(1)	-	Ser81Leu(1)	Ile218Thr+Ile219Phe(1)
ST579-CC10(1)	S(1)	-	Ser81Leu+Ala219Val(1)	Ile218Thr+Ile219Phe(1)
III(67)				
ST12-CC10(1)	N(1)		WT(1)	WT(1)
ST17-CC17(2)	N(1)	S(1)	Ser81Leu(2)	Ser79Phe(1), Gly128Asp+Ile218Thr+Ile219Phe(1)
ST19-CC19(60)	N(10), S(22)	N(10), S(18)	Ser81Leu(39), Ser81Leu+Ala219Pro(16), Ser81Leu+Ala219Val(1), WT(4)	Ser79Tyr(14), Ser79Phe(1), Ile218Thr(1), Ile218Thr+Ile219Phe(9), Ser79Tyr+Ile218Thr+Ile219Phe(7), WT(28)
ST23-CC23(1)	S(1)	-	Ser81Leu(1)	WT(1)
ST27-CC19(1)	N(1)	-	Ser81Leu(1)	Gln90His+His102Pro+Met108Rrg(1)
ST188-CC17(1)	S(1)	-	Ser81Leu(1)	Gly128Asp(1)
ST197-CC19(1)	S(1)	-	Ser81Leu(1)	WT(1)
V(9)				
ST19-CC19(9)	S(2)	N(1), S(6)	Ser81Leu(6), Ser81Leu+Ala219Pro(3)	Ser79Phe(2), Ile218Thr+Ile219Phe(2), WT(5)

*N, northern China; S, southern China; ST, sequence type; CCs, clonal complexes; FQ, fluoroquinolone; GBS, Group B Streptococcus; WT, wild type*.

In total, serotype III isolates were predominantly ST19 (89.6%, 60/67), while serotype Ib isolates belonged almost to ST10 (94.5%, 52/55). III/ST19 and Ib/ST10 isolates accounted for 43.5% (60/138) and 37.7% (52/138) of the FQ-resistant isolates, respectively. Two ST17 isolates were identified, both belonging to serotype III (Table 2).

### Molecular Profiles of Mutations in *gyrA* and *parC* Genes

Of the 138 FQ-resistant GBS isolates, 131 (94.9%) *gyrA* mutant isolates and 63 (45.7%) *parC* mutant isolates were identified.

A Ser81Leu substitution was found in 99.2% (130/131) of the *gyrA* mutant isolates. Ala219Pro substitution linked to Ser81Leu was regularly noted in ST10 and ST19 isolates (28.1%, 36/128), but it was not found in other sequence types. A single Ala219Pro substitution occurred exclusively in ST23, and no substitutions were found in ST12 (Table 2).

The *parC* mutations showed more variety than *gyrA* mutations. Among *parC* mutations, a Ser79Phe or Tyr substitution was dominant (76.2%, 48/63), followed by Ile218Thr and Ile219Phe double substitutions (49.2%, 31/63). Interestingly, ST10 isolates were identified with Ser79Phe (84%, 21/25), while ST19 isolates were limited to Ser79Tyr (95.7%, 22/23); 96.9% (31/32) of Ile218Thr substitutions were linked to Ile219Phe, and 100% (31/31) of the Ile219Phe substitutions were associated with Ile218Thr (Table 2). An infrequent Gly128Asp substitution was identified in ST17 and ST188 isolates, which belonged to CC17, and rare triple substitutions were noted in ST10, ST19, and ST27. Conversely, no substitutions were found in ST12, ST23, and ST197 isolates (Table 2).

### High Frequency of MDR Among FQ-Resistant GBS

FQ-resistant GBS isolates demonstrated considerable cross-resistance to erythromycin (81.9%, 113/138), clindamycin (67.4%, 93/138), and tetracycline (60.9%, 84/138), with a high frequency of MDR (89.1%, 123/138). Accordingly, a high prevalence of *ermB* (62.3%, 86/138) and *tetM* (48.6%, 67/138) genes was found, but a low frequency of *lnub* (15.9%, 22/138) gene was observed (Table 3). ST10 isolates that were resistant to erythromycin (98.1%, 53/54) and harbored *ermB* gene (98.1%, 53/54) were more prevalent than erythromycin resistance (68.9%, 51/74; *P* < 0.001) and *ermB* gene containing (37.8%, 28/74; *P* < 0.001) ST19 isolates, whereas ST19 isolates that were resistant to tetracycline (93.2%, 69/74) and carried *tetM* gene (81.1%, 60/74) were more common than tetracycline resistance (11.1%, 6/54; *P* < 0.001) and *tetM* gene containing (3.7%, 2/54, *P* < 0.001) ST10 isolates. All ST12, ST17, and ST188 isolates had *tetO* gene (Table 3).

**Table 3:** Prevalence of resistance phenotypes and genotypes among FQ-resistant GBS.

**ST-CCs (n)**	**Phenotypes (*****n*****, %)**			**Genotypes (*****n*****, %)**					
	**ERY^a^**	**CLI**	**TET^a^**	***gyrA***	***parC***	***ermB*^a^**	***lnuB***	***tetO***	***tetM*^a^**
ST10-CC10 (54)	53 (98.1)	46 (85.2)	6 (11.1)	54 (100)	26 (48.2)	53 (98.1)	6 (11.1)	3 (5.6)	2 (3.7)
ST12-CC10 (2)	2 (100)	2 (100)	2 (100)	0	0	2 (100)	0	2 (100)	1 (50)
ST17-CC17 (2)	2 (100)	2 (100)	2 (100)	2 (100)	2 (100)	2 (100)	1 (50)	2 (100)	0
ST19-CC19 (74)	51 (68.9)	38 (51.4)	69 (93.2)	69 (93.2)	32 (43.2)	28 (37.8)	14 (18.9)	11 (14.9)	60 (81.1)
ST23-CC23 (2)	2 (100)	2 (100)	2 (100)	2 (100)	0	0	0	0	2 (100)
ST27-CC19 (1)	1 (100)	1 (100)	1 (100)	1 (100)	1 (100)	0	0	0	1 (100)
ST188-CC17 (1)	1 (100)	1 (100)	1 (100)	1 (100)	1 (100)	0	0	1 (100)	0
ST197-CC19 (1)	1 (100)	1 (100)	1 (100)	1 (100)	0	1 (100)	1 (100)	0	1 (100)
ST597-CC10 (1)	0	0	0	1 (100)	1 (100)	0	0	0	0
Total (138)	113 (81.9)	93 (67.4)	84 (60.9)	131 (94.9)	63 (45.7)	86 (62.3)	22 (15.9)	19 (13.8)	67 (48.6)

*STs, sequence types; ERY, erythromycin; CLI, clindamycin; TET, tetracycline*.

*Fluoroquinolone (gyrA and parC), erythromycin (ermB), clindamycin (lnuB), and tetracycline (tetO and tetM)*.

a *Significant difference between ST10 and ST19 (P < 0.001). Differences between groups were assessed by chi-square test of proportions*.

### ICE Was Identified in a III/ST19 MDR Isolate by Sequencing

**Figure 1:**
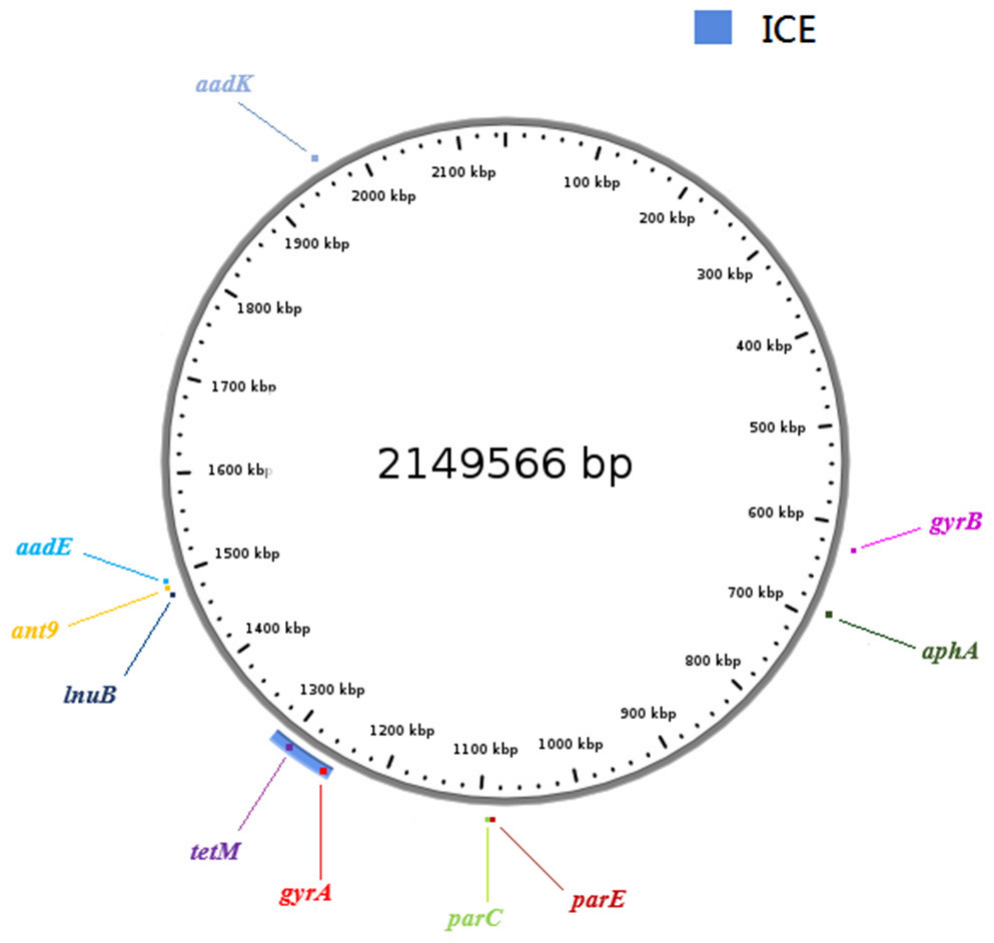
A comprehensive view of the new ICE and resistance genes in Group B *Streptococcus* (GBS) genome. The ring shows the genome length (kbp) of III/ST19, strain BJ01. The blue in the lower left corner represents the newly identified ICE. The locations of *tetM* and *gyrA* genes are marked, as well as the aminoglycoside resistance genes (*aadK, aadE, ant6, ant9*, and *aphA*) and other resistance genes in QRDR (*gyrB* and *parE*). ICE, integrative and conjugative element.

**Figure 2:**
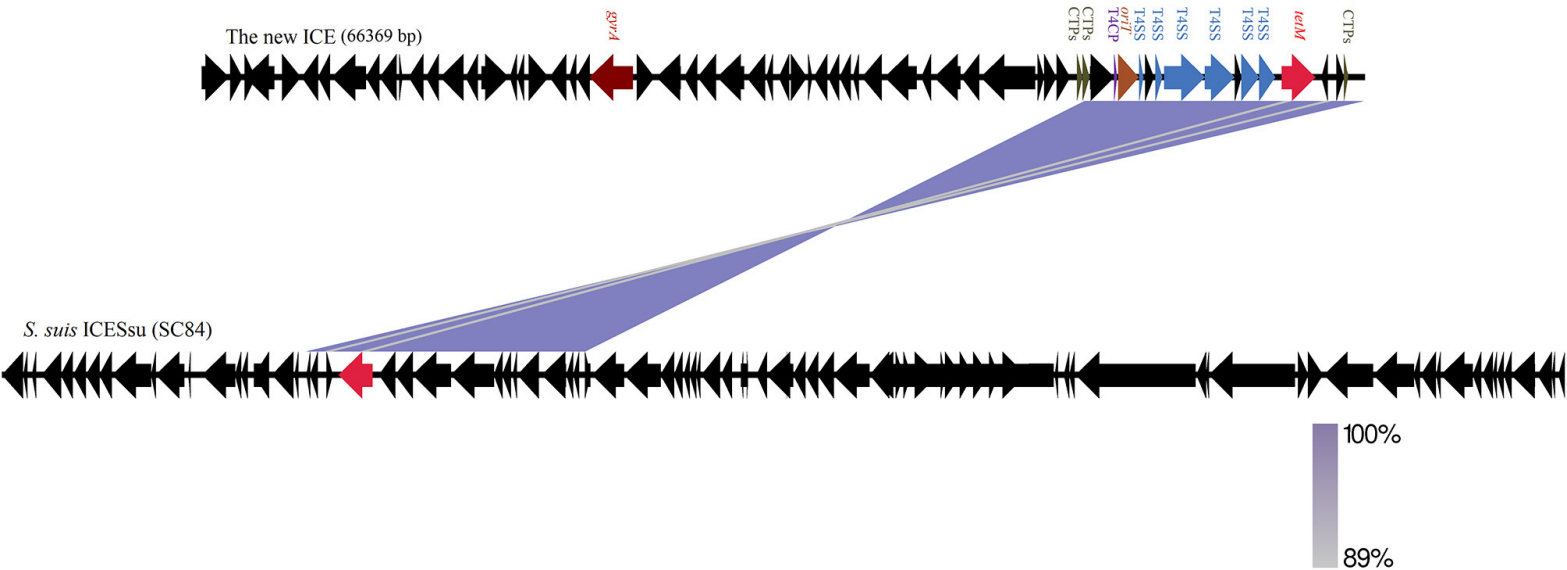
Schematic representation of the core structure of the new ICE and nucleotide sequence similarity to ICESsu (SC84) from *S. suis*. Coding sequences of the new ICE in the Group B *Streptococcus* (GBS) strain BJ01 isolate are depicted by arrows. Purple bands indicate BLAST matches between sequences in opposite orientations. The intensity of the colors indicates the strength of the match. ICE, integrative and conjugative element; T4SS, type IV secretion system; *oriT*, origin site of DNA transfer; T4CP, type IV coupling proteins; CTPs, conjugative transposon proteins.

Horizontal gene transfer events can lead to the exchange of genomic regions and are considered an important mechanism for the diversification of GBS (Brochet et al., 2008). A new ICE conferring antibiotic resistance to erythromycin and chloramphenicol was identified in a III/ST19 isolate, and their resistance phenotypes proved transferable (Morici et al., 2016). Therefore, we hypothesized that there may be putative MGEs in III/ST19 MDR isolates conferring antibiotic resistance. To verify this hypothesis, whole genome sequencing was performed on levofloxacin, clindamycin, and tetracycline resistance III/ST19 strains, which carry *gyrA, parC, lnuB*, and *tetM* genes, to look for potential MGEs. In recent years, a systematic approach has been used for the large-scale identification of candidate ICEs in genomic sequence databases. Regions that contained conserved features of conjugative elements, including genes predicted to encode conjugative relaxases, type IV coupling proteins (T4CP), and type IV secretion systems (T4SS), were identified in genomic sequences (Bi et al., 2012; Johnson and Grossman, 2015). In the present study, a novel ICE of 66,369 bp was identified in the genome, inserted at the 1,249,355 to 1,315,723 bp location, and harboring the *tetM* gene conferring tetracycline resistance and the *gyrA* gene, with a Ser81Leu mutation in the QRDR located 39,736 bp upstream of *tetM*. By comparing the comprehensive antibiotic resistance database, aminoglycoside resistance genes (*aadK, aadE, ant6, ant9*, and *aphA*) and other resistance genes in QRDR (*gyrB* with no mutation and *parE* with His225Tyr, Pro356Ser, and Ile507Val substitutions) were also identified in the BJ01 strain (Figure 1). No erythromycin resistance genes (*ermA, ermB*, or *mefA*) were found, which was consistent with the phenotype. The core genes of ICE contained 62 open reading frames (ORFs). The integrase was TIGR02224 and belonged to the tyrosine recombinase family, which recognized a 19-bp *att* sequence (TGCTGGTAAAACAACTTTT) at the 3′-end of *rplL* gene, a well-known hotspot for ICE integration in the *Streptococcus* spp. The relaxase was Rep_trans, and the T4CP was FtsK/SpoIIIE (Figure 2).

### Characterization and Organization of the New ICE

The ICE presented a typical mosaic organization composed of different modules that showed a high sequence similarity to other ICEs found in other *Streptococcus* spp., such as *Streptococcus suis, Streptococcus gallolyticus, Streptococcus pyogenes*, and *S. pneumoniae*. Among those in the ICEberg database (Bi et al., 2012), this novel ICE revealed the 99.41% nucleotide sequence similarity to *S. suis* ICESsu (SC84, nucleotide sequence ID: FM252031; Figure 2).

More detailed sequence analysis of the 62 ORFs displayed that the regions ORF51, ORF53 to ORF55, and ORF57 to ORF58, annotated as T4SS, played an important role in delivering protein and DNA substrates to target cells, generally by a contact-dependent mechanism. The type IV coupling proteins (T4CP) are an essential constituent of T4SS and lie in the region ORF49. The regions ORF46, ORF47, and ORF62, encoded conjugative transposon proteins involved in the movement from one bacterial cell to another by a process requiring cell-to-cell contact. The replication initiation factor occupied the region between 1,302,049 and 1,303,254 bp, with the 133 bp *oriT* region, and both were located in ORF50. ORF22 encoded the *gyrA* gene, conferring resistance to fluoroquinolone, and the *tetM* gene conferring tetracycline resistance was located in ORF59 (Figure 2).

## Discussion

Chinese mainland national epidemiological data on FQ-resistant GBS isolates from neonatal invasive infection and maternal colonization have not been well characterized. Our study showed that FQ resistance prevalence was 32.4%, close to the 37.7% reported by a previous study in Beijing, China (Wang et al., 2013). Notably, both of these percentages were markedly higher than reports from Europe and North America (0.2–1.5%) (Biedenbach et al., 2006; Hays et al., 2016), Taiwan (5.6%) (Wu et al., 2017), and Japan (non-susceptibility rate, 18.4%) (Kimura et al., 2012). One possibility for the high FQ resistance in mainland China is the widespread use of antibiotics in community-acquired infections and their extensive application in animal husbandry (Zhao et al., 2015; Zhu et al., 2019). Furthermore, the described FQ-resistant GBS isolates in previous reports were mainly ST19 (Wang et al., 2013; Hays et al., 2016), while ST10 accounted for a larger proportion in mainland China. Particularly, Ib/ST10 isolates were mainly distributed in northern China, while III/ST19 isolates were predominant in southern China. This finding is highly consistent in neonates and pregnant women, which strongly suggests the potential transmission of FQ-resistant GBS in local areas.

The main mutations identified in QRDR were a Ser81Leu substitution in the *gyrA* gene, and a Ser79Phe or Tyr substitution in the *parC* gene, as previously reported (Piccinelli et al., 2015; Hays et al., 2016; Wu et al., 2017). To our best knowledge, we believe that this is the first study to identify the novel mutation of the copresence of both Ile218Thr and Ile219Phe in the *parC* gene, and such a high frequency (96.9%) of these substitutions co-occurring implies that they are related. Further verification of gene function is needed to determine whether these two substitutions cause, or are associated with, FQ resistance. *Streptococcus* spp. have been suggested as the reservoirs of FQ-resistance genes, which can be transferred horizontally among *Streptococcus* spp., resulting in the spread of FQ resistance (Ferrándiz et al., 2000). Consider that some amino acid substitutions are associated with specific sequence types, which suggests that resistance to FQ was potentially acquired via horizontal clonal expansion. On the other hand, new mutation patterns have emerged and prevailed in certain isolates. Ib/ST10 isolates show high resistance to erythromycin and prominently possess the *ermB* gene, whereas III/ST19 isolates display high resistance to tetracycline and notably contain the *tetM* gene. These data indicate that there are two main lineages of clonal dissemination in FQ-resistant GBS isolates.

These findings, taken together with other reported literatures, have significant clinical implications focused mainly on the emergence of MDR GBS in clinical settings (Seki et al., 2015; Hays et al., 2016). Clinically significant antimicrobial resistance genes are usually located on different MGEs, mainly ICEs, which are highly variable and can easily lose or acquire different modules and, therefore, play a vital role in the acquisition and transmission of antimicrobial resistance genes (Wozniak and Waldor, 2010; Vrancianu et al., 2020). ICESag236 was identified in a III/ST19 isolate that harbored the macrolide resistance genes *mef* (I) and *erm*(TR) and chloramphenicol resistance gene *catQ* (Morici et al., 2016). In this study, a new ICE-containing tetracycline gene *tetM* and the *gyrA* gene in the QRDR was identified in a III/ST19 MDR isolate. Interestingly, the ICESag236 was originally identified in a III/ST19 pathogenic strain isolated from urine in a remote region of Italy, implying the horizontal transfer events of antimicrobial resistance genes harbored in putative ICEs, which is the likely cause of MDR in the III/ST19 lineage.

The first ICE discovered in GBS was ICESa2603, which is considered the prototype of the ICE family based on the integrase (Tettelin et al., 2002; Bi et al., 2012). It is able to retain its features of transferability among the *Streptococcus* spp. (Haenni et al., 2010; Huang et al., 2016). Subsequently, ICESa2603-like ICEs have been identified in clinical isolates (Davies et al., 2009), and to date, the ICESa2603 family and ICESa2603 family-like ICEs have been extensively studied in *Streptococcus* spp. Most of these encode a serine recombinase family integrase gene that specifically recognizes a 15-bp *att* sequence (TTATTTAAGAGTAAC) at the 3′-end of *rplL*. In this study, we identified the novel ICE encoding a tyrosine family integrase gene that specifically recognizes a 19-bp *att* sequence (TGCTGGTAAAACAACTTTT). Despite the difference in integrases, both types of ICEs are transmitted in the similar way, with chromosomes removed and a circular molecule formed (Johnson and Grossman, 2015). Homology searches of this novel ICE sequence shows high nucleotide sequence similarity (99.41%) to *S. suis* ICESsu. Additionally, an ICESag37 similar to *S. suis* SC070731 has also been identified in Shanghai, China (Zhou et al., 2017). These similarities suggest the universality of horizontal transfer of resistance genes among *Streptococcus* spp. in mainland China, resulting in a high isolation rate of MDR among FQ-resistant GBS. Further studies on conjugation and transmissible mechanisms of ICEs in these isolates will investigate the cause(s) of MDR through horizontal transfer of ICEs.

In conclusion, this multicenter study describes the molecular profiles of FQ-resistant GBS in mainland China. III/ST19 is more common in the south, while Ib/ST10 is more prevalent in the north. A new ICE conferring antibiotic resistance in a III/ST19 isolate was identified. This work highlights the significant molecular characteristics between northern and southern China and the high prevalence of MDR GBS in mainland China and emphasizes the need for continuous molecular-level surveillance geographically and further research to characterize the mechanisms involved in triggering ICEs transfer.

## Data Availability Statement

The datasets presented in this study can be found in online repositories. The names of the repository/repositories and accession number(s) can be found in the article/supplementary material.

## Ethics Statement

The studies involving human participants were reviewed and approved by The Medical Ethics Committee of Guangzhou Women and Children's Medical Center, Guangzhou, China (No. 2016050405). Written informed consent to participate in this study was provided by the participants' legal guardian/next of kin.

## Author Contributions

This study was conceived and designed by HL and WJ. The analyses and first draft of the paper were undertaken by KG and CG. Statistical analysis was performed by XG and QD. Microbiological techniques were carried out by LH, HZ, and YX. Tables and figures were prepared by LD, FG, and LZ. C-YC and DM provided critical comments on the draft and managed subsequent revisions. All authors reviewed and approved the manuscript.

## Conflict of Interest

The authors declare that the research was conducted in the absence of any commercial or financial relationships that could be construed as a potential conflict of interest.
